# Vasopressin and epinephrine in the treatment of cardiac arrest: an experimental study

**DOI:** 10.1186/cc6838

**Published:** 2008-03-14

**Authors:** Konstantinos Stroumpoulis, Theodoros Xanthos, Georgios Rokas, Vassiliki Kitsou, Dimitrios Papadimitriou, Ioannis Serpetinis, Despina Perrea, Lila Papadimitriou, Evangelia Kouskouni

**Affiliations:** 1 University of Athens, Medical School, Department of Experimental Surgery and Surgical Research, Agiou Thoma Street, Athens, Greece

## Abstract

**Background:**

Epinephrine remains the drug of choice for cardiopulmonary resuscitation. The aim of the present study is to assess whether the combination of vasopressin and epinephrine, given their different mechanisms of action, provides better results than epinephrine alone in cardiopulmonary resuscitation.

**Methods:**

Ventricular fibrillation was induced in 22 Landrace/Large-White piglets, which were left untreated for 8 minutes before attempted resuscitation with precordial compression, mechanical ventilation and electrical defibrillation. Animals were randomized into 2 groups during cardiopulmonary resuscitation: 11 animals who received saline as placebo (20 ml dilution, bolus) + epinephrine (0.02 mg/kg) (Epi group); and 11 animals who received vasopressin (0.4 IU/kg/20 ml dilution, bolus) + epinephrine (0.02 mg/kg) (Vaso-Epi group). Electrical defibrillation was attempted after 10 minutes of ventricular fibrillation.

**Results:**

Ten of 11 animals in the Vaso-Epi group restored spontaneous circulation in comparison to only 4 of 11 in the Epi group (*p *= 0.02). Aortic diastolic pressure, as well as, coronary perfusion pressure were significantly increased (*p *< 0.05) during cardiopulmonary resuscitation in the Vaso-Epi group.

**Conclusion:**

The administration of vasopressin in combination with epinephrine during cardiopulmonary resuscitation results in a drastic improvement in the hemodynamic parameters necessary for the return of spontaneous circulation.

## Introduction

Cardiac arrest affects more than 700,000 people per year in Europe [1-3]. Ventricular fibrillation (VF) is used to treat up to 40% of the cases when help arrives [4-6]. VF requires immediate bystander cardiopulmonary resuscitation (CPR) and electrical defibrillation [7].

The preferred drug for more than 100 years for use during VF has been epinephrine (adrenaline) [8]. Epinephrine's vasoconstrictive action results in a rise in the aortic pressure, thus increasing the coronary perfusion pressure (CPP) [9,10].

Vasopressin also has a vasoconstrictive action in the vascular network of the skeletal muscles, bowel, fat tissue, skin and, to a lesser degree, the coronary and renal vessels, while it causes vasodilation in the brain vessels. This results in an increase of the coronary perfusion pressure and, in general, an increase of blood flow to the vital organs without causing a dramatic increase in the myocardial oxygen consumption [11,12]. The aim of the present study is to assess whether the combination of vasopressin with epinephrine (Vaso-Epi combination) would increase initial resuscitation success demonstrated by the return of spontaneous circulation (ROSC).

## Materials and methods

After approval by the General Directorate of Veterinary Services, 22 Landrace/Large-White piglets of both sexes, all from the same breeder, with an average weight of 19 ± 2 kg were included in the study. Prior to any procedure, animals were randomized into two groups with the use of a sealed envelope indicating the animal's assignment to either the Epi group (11 animals; saline as placebo (10 ml dilution, bolus) + epinephrine (0.02 mg/kg)) or the Vaso-Epi group (11 animals; vasopressin (0.4 IU/kg/10 ml dilution, bolus) + epinephrine (0.02 mg/kg)). The study was blinded as to the medication used.

The experimental protocol has been described previously [13]. Briefly, anesthesia was induced with an intravenous bolus of propofol and the pigs were intubated with a 4.5 or 5 mm endotracheal tube (Portex, ID Smiths Medical, Keene, NH, USA). Additional propofol, cis-Atracurium and Fentanyl were administered immediately before connecting the animals to the automatic ventilator (ventiPac Sims pneuPac Ltd, Luton UK) with oxygen (FiO_2 _21%). Propofol infusion and additional doses of cis-Atracurium and Fentanyl followed. The animals were ventilated with the aid of a volume-controlled ventilator (total tidal volume 10 ml/kg). End-tidal CO_2 _was monitored (AG-400R, Nihon Kohden Italia, Bergamo, Italy) and the respiratory frequency was adjusted to maintain PETCO_2 _at 35 to 40 mmHg. Cardiac rhythm was monitored with an electrocardiogram (Mennen Medical, Envoy, Papapostolou, Athens, Greece).

Both of the internal jugular veins and the left carotid artery were prepared surgically. The systolic and diastolic aortic pressure were monitored continuously by inserting a normal saline-filled (model 6523, USCI CR, Bart Inc, Athens, Greece) arterial catheter into the descending thoracic aorta via the right common carotid artery. Both internal jugular veins were catheterized with a 6F sheath and a Swan-Ganz catheter (Opticath 5.5 F, 75 cm Abbott, Ethicon Mersilk™, Ladakis, Athens, Greece) was inserted into the right atrium for continuous measurement of systolic and diastolic right atrial pressure via the left jugular vein. The pressure was monitored using conventional external pressure transducers (Abbott Critical Care Systems, Transpac IV, Athens, Greece). CPP was calculated as the difference between diastolic aortic pressure and time-coincident mean right atrial pressure.

After allowing animals to stabilize for 40 minutes, baseline measurements were obtained and then a 5F flow-directed pacing catheter (Pacel™; 100 cm, St Jude Medical, Ladakis, Athens, Greece) was inserted through the right internal jugular vein into the apex of the right ventricle. VF was induced via a 9 V lithium battery. VF was confirmed electrocardiographically and in combination with the sudden drop of mean arterial pressure as described previously [13].

Immediately following confirmation of VF, mechanical ventilation and propofol infusion were ceased. Animals were left untreated for 8 minutes, representing the average time it takes for emergency medical services to arrive [14].

Resuscitation procedures were started by setting inspired oxygen concentration to 100%, followed by drug administration. All drugs were administered via the lateral auricular vein, thus simulating a peripheral vein via which drugs are administered in cardiac arrest victims in an emergency setting. Precordial compression began with a mechanical chest compressor (Thumper, Michigan instruments, Talon Court, SE, USA) for 2 minutes. Compressions were maintained at a rate of 100/minute. After 2 minutes of precordial compression, defibrillation was attempted with 4 J/kg monophasic waveform shock (Porta Pak/90-Medical Research Laboratories Inc, Buffalo Grove, IL, USA). In the case of failure to convert to a cardiac rhythm compatible with pulse, precordial compression was resumed for 2 minutes before the delivery of a second shock.

The endpoints were defined as ROSC, asystole or persisting VF after the third defibrillation attempt. ROSC was defined as the presence of an organized cardiac rhythm with a mean arterial pressure of at least 60 mmHg for a minimum of 5 minutes. The successfully resuscitated animals were monitored for 60 minutes while anesthesia was maintained. All animals were humanely killed by an intravenous overdose of thiopental (2 g).

The study was powered statistically to detect changes in ROSC. Data are expressed as the mean ± standard deviation (SD) for continuous variables and as percentages for categorical data. The Kolmogorov-Smirnov test was utilized for normality analysis of the parameters. Comparisons of continuous variables were analyzed using Student's *t*-test and the Mann-Whitney non-parametric test, as appropriate. Comparisons of categorical variables were analyzed using Fisher's exact test. Paired samples *t*-test and Wilcoxon tests were used for the comparison of different time measurement of parameters for each group. A comparison of the percentage change from baseline of the parameters during the observation period between two groups was made using the Mann-Whitney test. Moreover, using the analysis of covariance model the difference between groups was compared for all parameters at each time point controlling for baseline difference using the value of parameter at each time point as the dependent variable and baseline measurements as covariates.

Differences were considered as statistically significant if the null hypothesis could be rejected with >95% confidence (*p *< 0.05). All analyses were conducted using SPSS, version 13.00 (SPSS Inc, Chicago, IL, USA).

## Results

Baseline hemodynamic measurements did not differ between the two groups (Table 1). By the end of the eighth minute of VF, mean arterial pressure decreased from 89.3 ± 7.57 to 22.5 ± 3.31 mmHg in Epi group and from 89.0 ± 12.06 to 20.77 ± 3.96 mmHg in the Vaso-Epi group (*p *= 0.316). CPP declined rapidly and was 0.60 ± 0.96 mmHg in Epi group and 0.77 ± 0.83 mmHg in Vaso-Epi group (*p *= 0.675) during the eighth minute of untreated VF in both groups.

**Table 1:** Baseline variables in the two different groups

	**HR (bpm)**	**SAP (mmHg)**	**DAP (mmHg)**	**MAP (mmHg)**	**MRAP (mmHg)**	**CPP**
**Epi group**	108 ± 17	104 ± 8	78 ± 12	89 ± 8	11 ± 1	67 ± 11
**Vaso-Epi group**	123 ± 16	104 ± 12	82 ± 12	91 ± 16	11 ± 2	75 ± 13
*p*-value	0.084	0.968	0.479	0.948	0.777	0.193

CPP = coronary perfusion pressure; DAP = diastolic aortic pressure; HR = heart rate; MAP = mean aortic pressure; MRAP = mean right atrial pressure; SAP = systolic aortic pressure.

**Figure 1:**
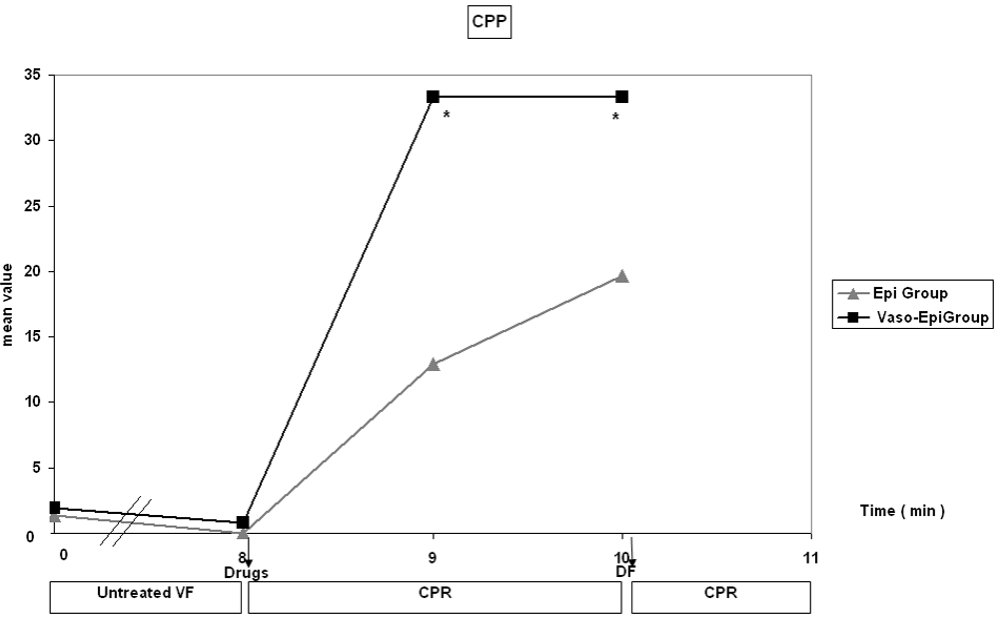
**Coronary perfusion pressure (CPP) fluctuation during the experiment**. DF = defibrillation; CPR = cardiopulmonary resuscitation (*p < 0.0001 Vaso-Epi group versus Epi group).

**Figure 2:**
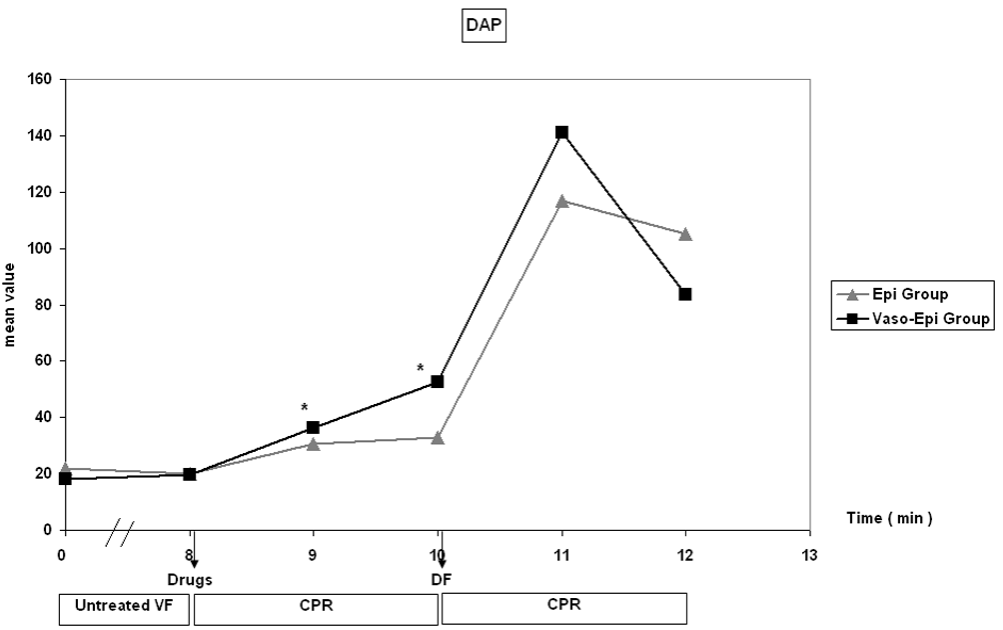
**Diastolic aortic pressure (DAP) fluctuation during the experiment**. DF = defibrillation; CPR = cardiopulmonary resuscitation (*p < 0.0001 Vaso-Epi group versus Epi group).

In the first minute of CPR, CPP rose significantly in the Vaso-Epi group and remained statistically higher in the second minute of CPR (Figure 1). A significant increase in diastolic aortic pressure was also noted between groups (Figure 2).

ROSC was observed in 4 animals in Epi group, while 10 animals achieved ROSC in the Vaso-Epi group (*p *= 0.02). More specifically, 4 animals in Epi group were successfully resuscitated after the first defibrillation and no further animals achieved ROSC in the following defibrillation attempts. In the Vaso-Epi group, 10 animals were resuscitated after the first defibrillation and 1 animal failed to achieve ROSC. This animal, without any external stimuli, presented with acute complete atrioventricular block, followed by non-sustained ventricular tachycardia and, finally, pulselles electrical activity. In this animal, an autopsy revealed pneumonia, whereas routine autopsy of the rest of the animals in both groups showed no evidence of pathology in the cardiopulmonary system. Furthermore, the total number of shocks in Epi group was 25 compared with 12 in Vaso-Epi group.

All animals that were resuscitated successfully were monitored for 1 hour. Table 2 summarizes the parameters measured during the 60th minute after ROSC. No statistically significant difference was found between the two groups during the whole post-resuscitation period.

**Table 2:** Parameters measured during the 60th minute after the return of spontaneous circulation

	**HR (bpm)**	**SAP (mmHg)**	**DAP (mmHg)**	**MAP (mmHg)**	**MRAP (mmHg)**
**Epi group**	146 ± 31	103 ± 24	77 ± 25	90 ± 26	16 ± 3
**Vaso-Epi group**	135 ± 17	88 ± 20	69 ± 14	78 ± 16	14 ± 4
*P*	0.440	0.287	0.495	0.26	0.541

DAP = diastolic aortic pressure; HR = heart rate; MAP = mean aortic pressure; MRAP = mean right atrial pressure; SAP = systolic aortic pressure.

## Discussion

In case of VF, the Advanced Life Support Guidelines of the European Resuscitation Council recommend the periodic use of epinephrine if two initial defibrillations have failed [15]. The use of a vasopressor is thought to be beneficial in cardiac arrest by improving cardiac and brain blood flow during CPR [16-18].

Epineprhine increases CPP via systemic arteriolar vasoconstriction, which maintains peripheral vascular tone and prevents arteriolar collapse [19]. Furthermore, during experimental and clinical cardiac arrest, endogenous catecholamine concentrations are extremely high (up to 170 times normal levels in an animal model of VF) [20]. Thus, evidence suggests that epinephrine may be helpful in CPR, especially in short-term survival [15,19].

Vasopressin, an endogenous peptide, is a potent vasopressor agent and has been shown to be beneficial in CPR. Via the V_1 _receptors, it stimulates the contraction of vascular smooth muscles, resulting in peripheral vasoconstriction and increased blood pressure. Via the V_2 _receptors, vasopressin possibly induces vasodilation [21-23]. Unlike epinephrine, it is resistant to the effects of acidosis [24,25]. Endogenous vasopressin levels were found to be higher in survivors of cardiac arrest than those who died [26-28].

However, the recent international literature is not very encouraging in the use of vasopressin as a single agent of choice for cardiac arrest. On a systematic review and meta-analysis of 1,519 patients with cardiac arrest from 5 randomized controlled trials, the results demonstrate that there is no clear advantage of vasopressin over epinephrine and that vasopressin should not be recommended on resuscitation protocols until more solid human data on its superiority are available [29].

In a multicenter trial, the effects of vasopressin were similar to those of epinephrine in the management of cardiac arrest and pulseless electrical activity (PEA) [17]. On the other hand, most of the porcine models of cardiac arrest give encouraging results. Biondi-Zoccai et al, in a meta-analysis including 33 animal studies, showed that vasopressin appeared to be superior to both placebo and epinephrine in VF cardiac arrest [30]. These seemingly contradictory findings may be explained by the fact that many of the studies do not take into account a subgroup analysis such as the distinction between VF, PEA and asystole. Another fact that should be taken into consideration is that many experimental models refer to asphyxial cardiac arrest, which implies a different mechanism of induction of cardiac arrest than electrical stimulation, and also leads to a severely hypoxic myocardium. All of these facts may suggest that the usual approach of pharmacological CPR management to administer identical drugs and dosages for patients with cardiac arrest caused by different factors may have to be reconsidered. Furthermore, it is possible that when the degree of ischemia is fundamental, as during asphyxia, or when advanced cardiac life support is prolonged, a combination of vasopressin with epinephrine may be beneficial [31]. Wenzel et al [17] also provided recent supported for this finding in a clinical trial where the Vaso-Epi patient subgroup had significantly higher ROSC and hospital discharge rates. This finding may indicate that the interactions among vasopressin, epinephrine and the underlying degree of ischemia during CPR may be more complex than was thought previously [17]. Even if the Vaso-Epi subset of patients in the aforementioned study was fortuitous, our data also show a stronger vasoconstrictive effect of the combination of the two drugs in comparison to epinephrine alone in the first minute of CPR with an increase of CPP. This increase was further attenuated in the second minute when diastolic aortic pressure and CPP are significantly increased.

There are experimental models indicating that an epinephrine-vasopressin combination works better [32-34]. A secondary analysis [17] of a clinical retrospective out-of-hospital cardiac arrest study [18] drew the same conclusions. In our study we have taken two points stated previously by other authors into consideration: first, that vasopressin has greater activity than epinephrine under the hypoxic and acidic conditions of a prolonged cardiac arrest [35]; and, second, that its V_2_-mediated vasodilatory effect could improve the end-organ hypoperfusion resulting by epinephrine and catecholamine stimulation [19].

The institution of effective external cardiac compressions restores a pressure gradient between the aorta and the right atrium with a return of blood flow [36]. Chest compressions appear to be the most important factor, both in human and animal studies, and even short interruptions decrease CPP dramatically. In previous studies, CPP has been found to be the key determinant for successful defibrillation in humans and various animal models [9].

The quality of chest compressions should not be overlooked in the interpretation of the results of clinical studies. Chest compressions in animal models are standardized and are usually delivered mechanically. On the other hand, chest compressions in clinical studies are usually of poor quality [37,38]. The large clinical vasopressin studies were performed before problems in out-of-hospital CPR quality were recognized, therefore this factor should also be taken into consideration in the interpretation of the clinical outcome in vasopressin studies.

The authors recognize several limitations in the interpretation of the present findings. The study was conducted on apparently healthy pigs and its direct application to human victims of cardiac arrest has yet to be addressed. Furthermore, between-species differences in the effects of vasopressin have not been evaluated in the present study. For example, there are different receptors in pigs (lysine vasopressin) and in humans (arginin vasopressin) [34]. In addition, the experimental animals were anesthetized and the potential interactions of the different agents were not assessed.

## Conclusion

Our study has demonstrated that the combination of epinephrine and vasopressin in the treatment of VF cardiac arrest improved perfusion pressures and short-term survival, in comparison to the single use of epinephrine. This study adds some evidence to the existing literature of the epinephrine-vasopressin combination benefits and further evaluation of these results should be undertaken in the future.

## Key messages

• The combination of vasopressin and epinephrine resulted in a statistically significant elevation of both diastolic aortic pressure and CPP during CPR.

• The combination of vasopressin with epinephrine during cardiopulmonary resuscitation resulted in a drastic improvement concerning the return of spontaneous circulation (91% versus 36%).

## Abbreviations

CPP = coronary perfusion pressure; CPR = cardiopulmonary resuscitation; PEA = pulseless electrical activity; ROSC = return of spontaneous circulation; VF = ventricular fibrillation

## Competing interests

The authors declare that they have no competing interests.

## Authors' contributions

KS participated in the study design and drafted the manuscript. TX participated in the study design and revised the manuscript critically for important intellectual content. GR participated in performing the surgical preparation of this model and has made substantial contributions to the design of this model. VK participated in the experimentation and collected the final data. DPa was responsible for animal welfare and performed autopsies on the animals. IS participated in the experimentation and collected the final data. DPe, LP and EK critically revised the manuscript.
